# Impaired Comprehension of Alternating Syntactic Constructions in Autism

**DOI:** 10.1002/aur.1348

**Published:** 2013-11-13

**Authors:** Melissa D Stockbridge, Francesca G E Happé, Sarah J White

**Affiliations:** Institute of Cognitive Neuroscience, University College LondonLondon, UK; Institute of Psychiatry, King's College LondonLondon, UK

**Keywords:** autism, language development, syntax, dative alternation

## Abstract

Individuals on the higher-functioning end of the autism spectrum have significant impairments in communication. Language delay can occur, particularly in syntactic or structural linguistic knowledge. However, classically observed semantic deficits generally overshadow these structural deficits. This research examined the potential effects on comprehension of dative expressions that exhibited syntactic alternation versus those that were restricted, whether in syntactic construction or through marked semantic differences in construction. Children with autism and matched neurotypical control participants were presented with a sentence battery of dative statements representing these variations in construction and were asked to display basic comprehension of the sentence meaning by identifying the recipient, or indirect object, of the dative verb. Construction, restriction, and semantic differentiation variables were analyzed for potential effects on the rate of accurate comprehension. Both groups performed with greater accuracy when dative expressions used a prepositional phrase than when the dative action was expressed in the syntax. The autism group performed more poorly when the dative expression could syntactically alternate than when it was restricted. These effects improve our knowledge of how children with autism understand alternating grammatical constructions. ***Autism Res*** *2014, 7: 314–321.* © 2013 International Society for Autism Research, Wiley Periodicals, Inc.

Individuals diagnosed with autism spectrum disorder (ASD) show significant impairments in social knowledge, difficulties in pragmatic communication, and social deficits in behavior and activities [Fine, Bartolucci, Ginsberg, & Szatmari, 1991 ], overshadowing language delay in syntactic or structural linguistic knowledge [Baron-Cohen, 2005; Geurts & Embrechts, 2008 ]. Some research has suggested that mastery of phonology and syntax appears to follow the same course of development as it does in neurotypical children, albeit accomplished at a slower rate in a proportion of individuals [Tager-Flusberg, 1981 ]. However, certain pervasive syntactic deficits have been observed [Philofsky & Fidler, 2007; Saldaña & Frith, 2007 ] and may continue into adolescence [Eigsti, Bennetto, & Dadlani, 2007 ], even in children with average intelligence [Eigsti & Bennetto, 2009 ]. These deficits often are attributed to more general information processing errors thought to characterize behavior observed in children with ASD across domains. Although it is known that typically developing children may use syntax from 2 years old [Naigles, 1990; Waxman, Lidz, Braun, & Lavin, 2009 ], relatively little research has been conducted that addresses fine-grained linguistic deficits (e.g. verb- or noun-specific constructions and parameters) or the relationships between these structural frameworks [Bartlett, Armstrong, & Roberts, 2005, p. 205] and general patterns of ability observed in children with ASD.

Evidence from patterns of impairment observed in linguistic performance of children with ASD may serve to strengthen more encompassing theories about the disorder. Further, examining access and comprehension of a fine-grain level of implied information within sentence structure presents a yet-unexplored degree of complexity within the domain of abilities of children with ASD and may better inform understanding of how structural comprehension impairments result in semantic and pragmatic deficits.

Multiple theories have been put forth to describe the broad pattern of impairment observed in children with ASD, both within and beyond the language domain. One account of the pattern of deficits apparent in children with ASD is weak central coherence [Frith, 2008; Happé, 1999; Happé & Frith, 2006 ]. Central coherence describes the cognitive tendency to distill generalizable, common information from diverse input. It can describe a spectrum of cognitive styles from those who show a “strong” preference toward this process, finding key qualities of information, to those who show a “weak” central coherence, focusing on the parts rather than the whole. Grammatical constructions and their comprehension require an amount of generalization from a single structure of a word [Chomsky, 1965; generalization hypothesis, Golinkoff, Hirsh-Pasek, Cauley, & Gordon, 1987; Naigles, 1990 ]. For a child with typical central coherence approaching a syntactic construction, the comprehension of that construction is highly informed by experience of the language's grammar, generalized to a degree across individual verbs and instances of usage. Generalization should result in increased practice and ability across syntactic structures that share similar (or even interchangeable) pragmatic import, such as the syntactic structures of dative verbs. However, autistic children with this deficit cannot generalize as liberally as do typically developing children [in abstract reasoning, Minshew, Meyer, & Goldstein, 2002; in word learning, Tek, Jaffery, Fein, & Naigles, 2008 ]. This leads one to predict that children with ASD will have a relatively impaired ability to process alternating grammatical constructions compared with neurotypical children, who appear to benefit from some degree of generalization across syntactic forms. A second account of impairment in ASD is one of executive dysfunction [Damasio & Maurer, 1978; Ozonoff, Rogers, & Pennington, 1991; Prior & Hoffmann, 1990; see Hill, 2004 for a review]. Executive function, or the management of attention and inhibition, is required for textual inferences. As children transition between sentences, new information must become the focus of comprehension. Further, new syntactic structures (new rules of order-defined word relationships) must be followed. Herein, an overcommitment to a single syntactic structure (occasionally referred to as “sticky attention”), particularly in terms of roles within word order, may play a role in the profile of language abilities that has been observed. A child who has overly committed to a single structure's interpretation, or a single “set,” may be less inclined to perform appropriate shifts needed to interpret a second similar structure. This leads one to predict that children with ASD will have a relatively impaired ability to address demands presented by syntactic changes and may be more likely to adhere to a single interpretation rule across all attempts at sentence comprehension.

Little is known about the relationship between the cognitive skills associated with successful comprehension and successful integration of language structure. To examine one fine-grained distinction between structural components of language and the potential effect of a language cognition disorder on a particular component relative to another, we employed dative expressions in which a subject, direct object, and indirect object are present, simple and malleable in syntactic and semantic dimensions. When syntax is changed within a sentence, there are two possible effects to the meaning of that sentence: (a) an alteration can leave the semantic import relatively constant or (b) alternation can signal a significant difference in semantic content and, thus, one structure is preferred. If the structural change results in no significant change to the meaning of the sentence, then the verb can be said to be “alternating” [Rappaport Hovav & Levin, 2008 ]. For example, an alternating dative verb would be “read,” as in “Toby read Jenny the book.” or “Toby read the book to Jenny.” However, if this structural change results in a significant change to the meaning, then the verb can be said to be “restricted” in its structure. For example, a restricted dative verb would be “kick.” This is because the sentence, “Danny kicked Becky the ball.” implies that Becky has received the kicked ball, whereas “Danny kicked the ball to Becky.” implies only that the ball was kicked toward Becky. The first sentence conveys that the action expressed is completed and successful (“Danny [caused] Becky [to have] the ball.”), whereas the second sentence only conveys that the indirect object is the goal of the action (“Danny [caused] the ball [to go to] Becky.”) [Green, 1974; Pinker, 1989 ]. One could imagine that, after this second sentence, the next sentence could be “Becky's attempt to get the ball was thwarted by John.” This new information fits better after the second sentence than the first, and this intuition supports the different interpretations of the two structures as representing a completed action in the first sentence and an action goal in the second sentence [Jackendoff, 1972 ]. Additional analyses of this structure have been proposed in the literature [thematic and action tier account, Anderson, 1971, Jackendoff, 1983; see Rappaport Hovav & Levin, 2008 for review]. Access and comprehension of this level of implied information within sentence structure represents a yet-unexplored degree of structural complexity in this population and presents a context within which to examine how structural comprehension impairments result in semantic and pragmatic deficits.

The distinction between alternating and restricted verbs within the dative case provides a context within which to consider the potential of the previously discussed accounts of impairment in children with ASD as they relate to patterns observed in comprehension of syntax. The syntax used in dative expressions follows either the double object (DO) construction, where the subject and indirect object appear in the sentence prior to the direct object (the first sentence in the above examples), or the prepositional object (PO) construction, where the indirect object is in a prepositional phrase after the direct object, usually following the preposition “to” or “for” (the second sentence in the above examples). Dative actions also can be expressed in a passive construction. Well-constructed passive dative sentences contain a prepositional phrase; however, that prepositional phrase contains the name of the otherwise acting subject (usually following the preposition “by”). Consider:

1. DO construction—Danny kicked Becky the ball.
2. PO construction—Danny kicked the ball to Becky.
3. Passive construction—Becky was kicked the ball by Danny.

Aside from how these structures may be appreciated differently in sentence comprehension, particularly by children with ASD, there is much debate as to whether the relationship among these constructions reflect a single meaning at a deep structural level [Larson, 1988 ] or are truly representative of different meanings [Pinker, 1989 ]. The apparent existence of restricted verbs supports the multiple meanings interpretation [Krifka, 2001 ]. In most contexts, the distinction between meanings in alternating constructions is functionally irrelevant because it can be assumed that a direct object heading in the direction of an indirect object will successfully arrive at its destination. However, it has been proposed that typical developmental acquisition of language constraints relies on sensitivity to semantic distinctions within verbs [Pinker, 1984 ]. Thus, while the distinctions being discussed may appear to be highly specific, the inability to employ linguistic constraints properly may reflect the inability to access this degree of detail, which may have profound ramifications within higher-order language ability. Although finer models for this language behavior have been proposed [Krifka, 1999; Pesetsky, 2007 ], the simple model proposed by Pinker [1989 ] provides sufficient resolution to address the question of whether a significant effect on comprehension is observed in children with ASD given alternating and restricted syntactic forms.

In this research, we examined children and adolescents with ASD for the potential comprehension effects of dative expressions using both DO construction and PO construction, as well as comprehension effects of expressions that exhibit syntactic alternation versus those that are restricted. Comprehension herein is operationally defined as the ability to identify the indirect object of a phrase, demonstrating access to the correct role structure implied by the verb. While this is not the most direct measure of sensitivity to the finite difference in meaning between these structures, it was unclear that even adult native speakers of English would possess an explicit awareness of the nuanced difference between prepositional and DO construction. Thus, a broader indicator of access to sentence meaning was chosen. We tested three hypotheses: (a) Children with ASD will exhibit decreased comprehension of dative sentences compared with neurotypical counterparts matched for verbal competency across all conditions. This follows from the observation of structural language delay and syntactic difficulties noted in Tager-Flusberg [1981 ] and Philofsky and Fidler [2007 ]; (b) PO constructions will be associated with higher mean comprehension rates than DO constructions because of the presence of the word “to” that marks the recipient of the verb effect more explicitly; and (c) Children with ASD will exhibit lower comprehension rates on alternating constructions than they will on restricted constructions, while neurotypical children will not experience a significant effect of this variable. If children with ASD have weak central coherence, as discussed above, one would expect that they would have more difficulty distilling a rule from the dative sentence in order to comprehend what is meant. In alternating sentences, the neurotypical children likely benefit from their central coherence skill and, recognizing that the semantic import among PO and DO sentences often is irrelevant to accurate comprehension, will be able to generalize across conditions. However, within the restricted condition, this rule generalization has less potential to aid comprehension. Thus, one would predict that children in the ASD group would perform relatively better in the restricted condition. These hypotheses reflect the predicted relationships among the three variables present in this experiment: group, construction, and restriction.

## Methods

### Participants

Ethical approval for the study was received from the University College London Research Ethics Committee, and consent was obtained from the parents of all participants prior to inclusion in the study. Eighteen participants with ASD, aged 7–16, were similar to 18 neurotypical control participants for gender (χ^2^(1, *N* = 36) = 3.01, *P* = 0.083) and British Picture Vocabulary Scale (BPVS) raw scores (*t*(34) = 0.863, *P* = 0.394) (see Table 1).

**Table 1:** Means (and Standard Deviations) for Participant Characteristics in Both Groups

	Children with ASD	Control children
*N* (male : female)	18 (14:4)	18 (9:9)
Age (years)***	12.51 (2.75)	8.31 (1.54)
Verbal IQ**	77.17 (19.37)	98.39 (14.86)
BPVS-II raw score	87.06 (27.30)	80.00 (21.39)
BPVS-II age equivalent	9.088 (3.33)	8.13 (2.82)
WISC-III UK forward count	7.33 (1.82)	8.17 (2.04)
WISC-III UK backward count	3.61 (1.94)	3.67 (1.19)
WISC-III UK digit span scaled score***	6.28 (3.14)	9.78 (2.13)

** *P* < 0.01;

*** *P* < 0.001.

ASD, autism spectrum disorder; BPVS-II, British Picture Vocabulary Scale, Second Edition; IQ, intelligence quotient; WISC-III UK, Wechsler Intelligence Scale for Children—Third Edition UK.

Children with ASD came from special schools with autism provision, while control participants were recruited from mainstream schools around London, UK. Following Philofsky and Fidler [2007 ], diagnosis on the autism spectrum was determined via prior clinical opinion. Children in the neurotypical control group had no known cognitive delays or current developmental diagnoses.

### Materials and Procedure

Verbal intelligence and working memory difficulty were assessed prior to administering the experimental battery. All tests were carried out during a single session. The BPVS, Second Edition [Dunn, Dunn, Whetton, & Burley, 1997 ] was administered to quantify receptive vocabulary as a measure of verbal intelligence. Raw scores were the basis of the experimental and control group matching [Mervis & Klein-Tasman, 2004 ]. The age range was selected to minimize confounding novel dative construction, which is observed in neurotypical children of chronological age 2–3 years [Conwell & Demuth, 2007; Gropen, Pinker, Hollander, Goldberg, & Wilson, 1989 ]; any children with a verbal mental age equivalent to 4 years old or below were therefore excluded from both groups. As the ASD group was of below-average intelligence quotient (IQ), group differences in chronological age and the standardized BPVS verbal intelligence score were inevitably significant in order to match the groups on BPVS raw score (age: *t*(34) = 5.670, *P* < 0.001; verbal IQ: *t*(34) = 3.688, *P* = 0.001).

The Digit Span subtest of the Wechsler Intelligence Scale for Children—Third Edition UK [Wechsler, 1992 ] was administered to address the contribution of working memory difficulty to task performance. While scaled scores on the assessment were significantly different (*t*(34) = 3.914, *P* < 0.001), these scores were again compromised by age differences between the groups. Thus, raw score digit span for the forward and backward portions of the assessment were considered separately, and group differences were not significant for forward (*t*(34) = 1.296, *P* = 0.204) or backward digit span (*t*(34) = 0.103, *P* = 0.918). Working memory difficulties could therefore not explain any group differences.

The experimental data collection consisted of an 80-sentence battery (see Table 2). For all sentences, the affected object was a familiar noun of one or two syllables, and the proper names used were controlled for phonological and syllabic complexity: vowels (V) and consonants (C) in either V-C-V or C-V-C-V patterns. Restricted and alternating verbs all reflected the dominant part of speech and did not differ in verb frequency per million words (*P* = 0.161) or on relative dominance of verb form to noun form (*P* = 0.766), based on SUBTLEX-US American English subtitles corpus of word frequencies [Brysbaert & New, 2009 ]. Gender effects also were controlled in the proper names. Mean length of utterance for alternating and restricted conditions were equal 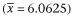. Mean length of utterance for PO 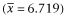 and DO 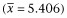 conditions were significantly different, with PO construction associated with approximately one additional word (the “*to*”). Mean length of utterance for passive sentences was necessarily longer 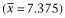, accounting for the grammatically necessary “*was*” and “*by*.” Sixteen dative sentences were presented that equally represented both the PO and DO constructions, both restricted (R) and alternating (A). Alternating verbs were presented in both constructions across participants, and variables were presented in randomized blocks using E-Prime 2.0 (Psychology Software Tools, Pittsburgh, PA). Each dative sentence was presented on a computer screen. At the participant's cue, the sentence was replaced by a question. The question asked the participant to identify the indirect object of the sentence. For example, “Becky threw the ball to Jamie” was followed by either “Who was thrown the ball?” or “Who was the ball thrown to?”^1^ Question construction was randomized across trials. Participants had to select either “Becky” or “Jamie” by pressing a key below the answer. Sixteen well-formed dative passive constructions of previously seen lexical items were also used as a control condition. A previously recorded reading in British English of each of the 80 sentences and questions played concurrently as the text appeared on the screen. Correct answers and time to response were measured.

**Table 2:** Stimulus Sentence Types and Distribution

	Children with ASD	Control children
Alternating double object	0.618 (0.165)	0.652 (0.224)
Restricted double object	0.640 (0.218)	0.611 (0.287)
Alternating prepositional object	0.664 (0.224)	0.779 (0.151)
Restricted prepositional object	0.761 (0.178)	0.803 (0.159)
Passive	0.518 (0.179)	0.476 (0.152)

ASD, autism spectrum disorder.

## Results

Data were computed using a 2 × 2 × 2 three-way mixed analysis of variance design (group × restriction × construction). Every participant in both groups completed all of the tasks presented. Mean accuracy levels across conditions are summarized in Table 3. Reaction time measures produced no significant results.

**Table 3:** Mean Accuracy Rates Across Conditions for Each Group (and Standard Deviation)

Sentence type	Sample sentence
Alternating double object (DO-A)	Sally read Harry the book.
Alternating prepositional object (PO-A)	Harry read the book to Sally.
Restricted double object (DO-R)	Gary bought Laura the puppy.
Restricted prepositional object (PO-R)	Lily drove the truck to Toby.
Passives (control)	Joey was shown the mouse by Sarah.

The main effect of construction on the accuracy rates of sentences showed significance (DO: 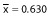, s = 0.034; PO: 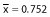, s = 0.027; *F*(1,34) = 21.76, *P* < 0.001, η_P_^2^ = 0.39). There were no significant main effects of group (ASD: 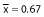, s = 0.039; control: 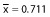, s = 0.039; *F*(1,34) = 0.537, *P* = 0.469) or restriction (A: 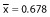, s = 0.028; R: 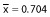, s = 0.030; *F*(1,34) = 1.975, *P* = 0.169).

A, alternating; DO, double object; PO, prepositional object; R, restricted.

There was a significant main effect of construction on the accuracy rates of sentences (DO: 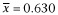, s = 0.034; PO: 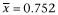, s = 0.027; *F*(1,34) = 21.76, *P* < 0.001, η_P_^2^ = 0.39), with PO being performed better than DO. There were no significant main effects of group (ASD: 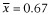, s = 0.039; control: 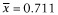, s = 0.039; *F*(1,34) = 0.537, *P* = 0.469) or of restriction (A: 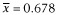, s = 0.028; R: 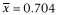, s = 0.030; *F*(1,34) = 1.975, *P* = 0.169).

**Figure 1:**
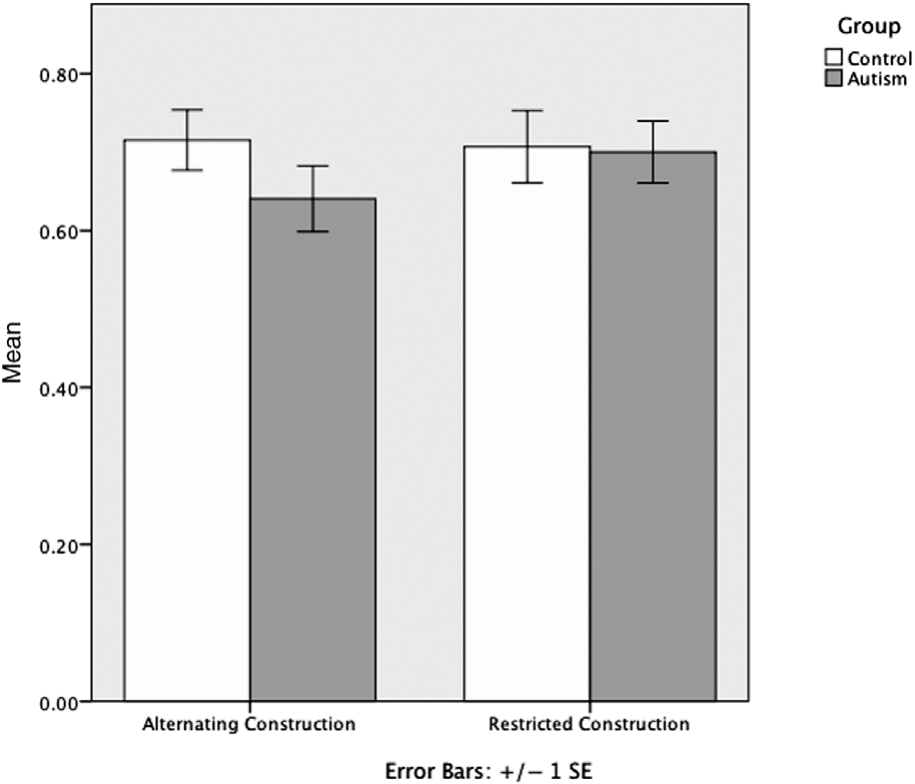
Differences in performance on alternation and restriction by group; error bars represent standard errors. There was a statistically reliable difference between the alternating (A: 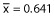, s = 0.178, 95 percent confidence interval (CI_95_) = 0.552–0.729) and restricted (R: 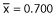, s = 0.167, CI*_95_* = 0.617–0.784) conditions seen in the autism spectrum disorder (ASD) group (*t*(17) = 2.053, *P* = 0.028), with better performance in the restricted condition. There was no difference in performance on the alternating or restricted conditions in the control group (A: 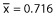, s = 0.163, CI*_95_* = 0.634–0.797; R: 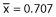, s = 0.195, CI*_95_* = 0.610–0.814; *t*(17) = 0.381, *P* = 0.354).

The interaction effect between group and restriction approached significance with a moderate effect size (Fig. 1; *F*(1,34) = 3.482, *P* = 0.071, η_P_^2^ = 0.093). This nearly significant result reflected an a priori hypothesis and was examined further in post hoc analysis. A one-tailed paired-samples *t*-test revealed a statistically reliable difference between the alternating (A: 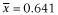, s = 0.178) and restricted (R: 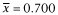, s = 0.167) conditions within the ASD group (*t*(17) = 2.053, *P* = 0.028), with better performance in the restricted condition. In the control group, there was no difference in performance on the alternating and restricted conditions, however (A: 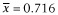, s = 0.163; R: 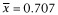, s = 0.195; *t*(17) = 0.381, *P* = 0.354). There was no significant interaction effect between group and construction (*F*(1,34) = 2.087, *P* = 0.158), between restriction and construction (*F*(1,34) = 2.228, *P* = 0.145), or among group, restriction, and construction (*F*(1,34) = 0.012, *P* = 0.915). Passive sentences produced no significant results between children with ASD (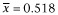, s = 0.179) and controls (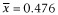, s = 0.152), with neither group performing significantly above chance.

## Discussion

This research examined three hypotheses concerning the comprehension of dative constructions by children with ASD when compared with children without developmental disorder or delay. First, children with ASD would perform comprehension tasks with less accuracy than neurotypical children. Second, children in both groups would be more accurate in response to sentences in which the structure included the explicit preposition “to” (PO construction) when compared with those sentences in which the structure and word order alone informed their decision (DO construction). Third, children with ASD would exhibit higher comprehension rates on those verbs that only can be presented in one construction (restricted) compared with those that can alternate without a significant change in meaning. We found that, in general, children with ASD did not perform significantly worse than their neurotypical peers across experimental conditions. However, children in both groups did perform better with sentences that used the PO construction, and children with ASD performed better with sentences that could only be presented in one construction than with sentences that could alternate.

This research provided an opportunity to examine the relationship between the ability to interpret syntax and the ability to interpret semantic content without changing the scale of the stimulus presented. Here, presumably as single-sentence stimuli were presented, no significant effect on semantic understanding across the dative sentence conditions was observed, indicating that at least at the single-sentence level semantic processing is intact in autism. The absence of a main effect of group is of particular interest because it raises a question about how assessments of syntax compare to the means by which higher-order language skills are assessed.

Indirect objects in sentences with PO construction were significantly more likely to be correctly identified than in the DO construction, perhaps because syntactic structure of PO constructions is intrinsically easier to manipulate or because of the ease with which some comprehension rule is correctly applied. This effect corroborates previous findings in an elicited repetition paradigm examining English dative alternation [Conwell & Demuth, 2007 ]. Passive sentences used as a control condition were designed to account for the possibility of a circumventing strategy; however, the results of this measure were inconclusive, as they were associated with the highest error rate, performing at chance level [a finding also reported in Caplan & Hildebrandt, 1988 ]. In the absence of significant findings across the measured parameters (raw BPVS, verbal age equivalence, digit span (forward, backward, and combined), chronological age, and standardized BPVS), it is impossible to make inferences regarding the performance observed. The lack of correlation with digit span provides some evidence that the absence of observed effect was not purely one due to working memory differences.

It is possible that some participants in one or both groups were circumventing the task with a rule less easily applied to DO constructions and that this contributed to the main effect in construction. If both groups were equally employing a rule across all conditions, one would not have expected to see any difference across the restriction conditions, yet the observed effect of restriction in the ASD group was significant. Perhaps the most parsimonious explanation for this effect is that the presence of the preposition “to” in the PO construction, signaling the direction of the direct object's transfer to the indirect object, makes that relationship easier to understand than one that must be inferred from the syntactic roles in the sentence [O'Grady, 1987, p. 63]. The performance in PO construction may be affected by frequency [Demuth, Machobane, Moloi, & Odato, 2005 ], as the PO form has been demonstrated to be more frequent in English usage [Gropen et al., 1989 ]. Also possible is the effect of the proper noun on the sentence, given the observed tendency to place longer, phonologically heavier objects last [e.g. Wasow, 2002 ]. Accuracy rates with DO construction and PO construction did not show significant effects of group, supporting the idea that interpreting syntactic roles without a lexical cue is not a skill that particularly is affected adversely by the difficulties in language associated with ASD.

The third hypothesis was that children in the ASD group would exhibit lower accuracy rates when presented with sentences containing verbs that could alternate between DO and PO constructions versus those that were restricted to one construction or the other. We found that children with ASD performed more poorly on the alternating than on the restricted constructions, while children in the control group performed similarly in both conditions. Correlation between working memory (digit span) tasks and performance on the various experimental conditions did not yield significant results. It is possible that this measure was confounded by the task difficulties encountered by some members of the ASD group when confronted with the requirement to name digits backward. The performance decrease in children with ASD when confronting alternating sentences could be explained by considering the proposed local processing bias, or bias toward attention to details in the absence of generalization [Happé & Frith, 2006 ]. If the children with ASD were unable to generalize that the correct response was to identify the indirect object of the sentence, then each stimulus would have been independently considered de novo. Thus, one might expect ASD participants to perform poorly on the alternating construction because presumably they were not as aided in their responses by the repetition of the lexical inventory as were children in the control group. This effect was far less relevant in the restricted conditions because the semantic content of the sentences in the restricted conditions was presented only once. The global processing bias in normally developing children might allow them to recognize that the 80-sentence battery was one in which the unifying concept was to identify the indirect object of whichever sentence was presented, allowing them to respond accurately regardless of the syntactic manipulations.

This research sought to examine language abilities in children with ASDs that transcended the levels of syntax and semantics. In order to bridge an apparent gap in the existing research, we observed the potential effects on comprehension of dative expressions that exhibited syntactic alternation versus those that were restricted, whether in syntactic construction or because of marked semantic differences in construction. Taken together, the observed effects support the interpretation that the syntactic effects present in these tasks overshadowed the potential semantic deficits and allowed children with ASD to perform at levels not significantly different from neurotypical children. This research does appear to lend support to the central coherence theory of ASD [Happé & Frith, 2006 ] and the notion that, while a central coherence deficit is not necessarily relevant to interpretation of a given lexical item, it is relevant *within* complex sentence structures as well as among sentences presented in a longer text. Appropriate further directions to refine the understanding of these effects would include an examination of syntax comprehension, like that assessed in this design, presented in paragraphs of text alongside higher-order pragmatic questions. Stimuli in such a design could resemble those used in the “strange stories” [Happé, 1994 ] with additional syntactic questions to be used together with these passages. Also, vignettes or image pairs could be used to examine subconsciously employed semantic differentiation between the prepositional and DO dative constructions. A design of this nature would reflect our finding that semantic differentiation among dative forms is something that neither ASD nor neurotypical children are consciously aware, at least in the literal terms proposed by theoretical psycholinguistics. Continued exploration of these effects on language comprehension in ASD still promises to provide improved intervention strategies or, potentially, increased diagnostic resolution for these children.

## Footnotes

1 These forms, in contrast to prescriptive constructions including the word “whom,” were used in order to better reflect the descriptive grammar of the target population.
